# The Journal

**Published:** 1851-11

**Authors:** 


### ARTICLE II.

### THE JOURNAL.

We are exceedingly sorry that we did not print enough of the first number of this volume to supply all our new subscribers. Many of them who have not received the Journal until now will understand the reason of our requiring them to begin in the middle of the volume, to be a want of back numbers.

The Intelligencer is issued regularly every other month and sent to subscribers who have paid up all arrearages at the the time of sending out each number.

The rule in reference to the Intelligencer cannot be varied from as it would lead to much trouble and defeat one of the objects of its publication. We wish all of our subscribers would avail themselves of the opportunity to get two Medical papers instead of one, without any additional charge.

We shall be obliged, soon, to make advance payment the only condition on which we send the Journal to subscribers.

